# Distribution of FDG-avid nodes in esophageal cancer: implications for radiotherapy target delineation

**DOI:** 10.1186/s13014-016-0731-6

**Published:** 2016-11-25

**Authors:** Brandon Garcia, Karyn A. Goodman, Lajhem Cambridge, Mark Dunphy, Abraham J. Wu

**Affiliations:** 1 Columbia University College of Physicians and Surgeons, New York, NY USA; 2 Department of Radiation Oncology, Memorial Sloan Kettering Cancer Center, 1275 York Ave, Box 22, New York, 10065 NY USA; 3 Molecular Imaging and Therapy, Memorial Sloan Kettering Cancer Center, New York, NY USA

**Keywords:** Esophageal neoplasms, Radiation oncology, Adenocarcinoma, Carcinoma, Squamous cell, Lymph nodes

## Abstract

**Purpose:**

Clinical target volumes (CTV) for radiotherapy (RT) in esophageal cancer (EC) are based on standard expansions of primary tumor volume. Data is needed to define regions at highest risk for occult disease, based on histology and location of the primary tumor. We therefore reviewed PET scans in EC patients to characterize the location of FDG-avid lymph node metastases (LNM).

**Materials and methods:**

We identified 473 EC patients with reviewable pre-treatment PET-CT scans. Tumors were classified by histology and location; 85% were distal or GE junction tumors and 71% were adenocarcinoma. FDG-avid LNM were classified using standard radiographic nodal atlases, and distances from primary tumor to paraesophageal LNM were also measured.

**Results:**

The most common LNM in upper EC were supraclavicular, retrotracheal and paratracheal. The most common LNM in lower EC were paraesophageal and in the gastrohepatic space. Overall, 55% of paraesophageal LNM were adjacent to primary tumor. Of upper esophageal tumors with paraesophageal LNM, 87% were adjacent to the tumor and none were >6 cm from tumor. However, 57% of lower esophageal tumors with paraesophageal LNM had non-adjacent paraesophageal nodes, 24% of which were >8 cm from the tumor.

**Conclusion:**

A more data-driven and individualized approach to CTV delineation could improve the therapeutic ratio of RT in esophageal cancer. These results can guide CTV delineation by indicating the potential distribution of nodal involvement in esophageal cancer.

**Electronic supplementary material:**

The online version of this article (doi:10.1186/s13014-016-0731-6) contains supplementary material, which is available to authorized users.

## Introduction

An estimated 482,300 new cases of esophageal cancer occured worldwide in 2014, with 18,170 in the United States. Because of the extensive network of esophageal lymphatics, metastasis to regional lymph nodes occurs early and frequently, contributing to an 85% mortality rate. Five-year survival for all patients with esophageal cancer over the past decade is estimated at 19%, which is significantly improved since the 1970s but still leaves much to be desired [1].

Both neoadjuvant and definitive chemoradiotherapy have become well-established standards of care in the treatment of esophageal cancer (EC) [2–4]. Currently, radiation treatment (RT) fields for EC are quite large as they are based on simple geometric expansions of the primary tumor volume to limit locoregional lymph node metastasis (LNM). For example, current clinical trials typically define the clinical target volume (CTV) by calling for a 4 cm longitudinal expansion and 1.5 cm radial expansion from the gross tumor volume (GTV) [5, 6].

However, data from esophageal squamous cell cancer has generated controversy regarding the effectiveness of an expanded CTV in preventing either local recurrence or LNM, and it is unclear whether such large and standardized fields are always necessary, including for esophageal adenocarcinomas [7–9]. A more tailored CTV limited to nodal stations at significant risk could reduce adverse effects of RT, but data are lacking to guide the choice of which lymph node regions to include, particularly for distal esophageal adenocarcinomas.

FDG-PET scans are now standard in the staging of esophageal cancer because of their ability to detect metastatic disease, including lymph node metastases (LNM). Several studies have demonstrated correlation between LNM indicated on staging PET scans and actual LNM based on surgical pathology [10, 11]. Therefore, we reviewed initial staging PET scans in patients with newly diagnosed esophageal cancer to characterize the frequency and location of FDG-avid LNM relative to the primary tumor and radiographic definitions of nodal stations.

## Materials and methods

Institutional review and privacy boards approved this study, and patient confidentiality was maintained as required by the Health Insurance Portability and Accountability Act. We then queried institutional databases to identify all patients receiving preoperative or definitive-intent RT at our institution for esophageal or gastroesophageal junction cancer between June 2007 and January 2014. All patients had confirmation of esophageal adenocarcinoma or squamous cell carcinoma on central pathologic review. We excluded patients with non-squamous or non-adenocarcinoma histology, distant metastases, synchronous malignancies, or who did not have a reviewable PET-CT scan prior to initiation of therapy. In cases where patients had more than one PET-CT prior to treatment, we analyzed the most recent one. In total, 473 patients who met these criteria were analyzed.

Tumor location was classified according to the geometric center of the FDG-avid lesion into the following categories: cervical (superior to the thoracic inlet); upper mediastinal (between the thoracic inlet and carina); lower mediastinal (between the carina and the gastroesophageal (GE) junction); and gastroesophageal (any tumor extending into the GE junction).

Most patients (58%) had PET-CT performed at our center. In these patients, PET-CT data were acquired on GE® Discovery PET-CT scanners, and PET images were reconstructed using an ordered subset expectation maximization (OSEM) iterative algorithm. CT data were acquired with 140 kVp and 70 mA, without IV contrast.

All FDG-avid lymph nodes described in the PET-CT report as at least “suspicious” (>50% probability of being malignant, according to our institutional standards for radiology reporting) were recorded and classified using standard nodal atlases: the International Association for the Study of Lung Cancer system for thoracic nodes, and the Japanese Research Society for Gastric Cancer system for abdominal nodes [12, 13]. The presence or absence of FDG-avid LNM in each of 28 nodal stations (12 thoracic and 16 abdominal) was recorded for each patient.

The rationale for large longitudinal CTV margins is based, in part, on concern for occult paraesophageal nodal spread from the primary tumor. Therefore, we also measured and recorded the longitudinal distance between the primary tumor and any FDG-avid LNMs arising in a paraesophageal location (thoracic stations 1, 2, 3P, 4, 7, and 8).

## Results

Median patient age was 66 years (range, 28–95), 76% were male, and 87% were Caucasian. Three-quarters had stage III tumors, predominantly adenocarcinoma (71%), with 89% classified as moderately or poorly defined. Lower thoracic (LT) tumors, defined as being inferior to the carina, comprised 88% of all tumors (40% lower mediastinal, 48% gastroesophageal), and were 80% adenocarcinomas, while upper thoracic (UT) tumors were 91% squamous cell carcinoma. Patient characteristics are summarized in Table 1.

**Table 1:** Patient characteristics

	Total (*n*)	%	With FDG-avid LN metastasis	%	Metastasis rate †
Total patients	473		204		43
- Male	361	76	163	80	45
- Female	112	24	41	20	36
Age at diagnosis					
- 20–49	38	8	22	11	58
- 50–59	103	22	44	22	43
- 60–69	156	33	70	34	45
- 70–79	125	26	50	25	40
- 80+	51	11	18	9	35
Median (range)	66 (28–95)		64 (28–88)		
Race					
- White	413	87	178	87	43
- Black	24	5	9	4	36
- Asian	18	4	9	4	50
- Other/Not specified	18	4	8	4	44
Tumor stage					
- T1	22	5	9	4	41
- T2	59	12	18	9	31
- T3	345	73	148	73	43
- T4	19	4	12	6	63
- n/a	28	6	17	8	61
Tumor differentiation					
- well-differentiated	7	1	3	1	43
- moderately-differentiated	219	46	95	47	43
- poorly-differentiated	205	43	94	46	46
- not specified	43	9	12	6	29
Tumor histology					
- Adenocarcinoma	338	71	138	68	41
- Squamous Cell	135	29	66	32	49
Tumor location					
Upper Thoracic	57	12	31	15	54
Cervical	15	3	3	1	20
- Adenocarcinoma	1	7	1	33	100
- Squamous Cell	14	93	2	67	14
Upper Mediastinal	42	9	28	14	67
- Adenocarcinoma	4	10	2	7	50
- Squamous Cell	38	90	26	93	68
Lower Thoracic	417	88	173	85	41
Lower Mediastinal	188	40	73	36	39
- Adenocarcinoma	124	66	46	63	37
- Squamous Cell	634	34	27	37	42
Gastroesophageal	229	48	100	49	44
- Adenocarcinoma	210	92	89	89	42
- Squamous Cell	19	8	11	11	58

†Metastasis rate is the percentage of total patients in a given category with FDG-avid LNM

**Fig. 1:**
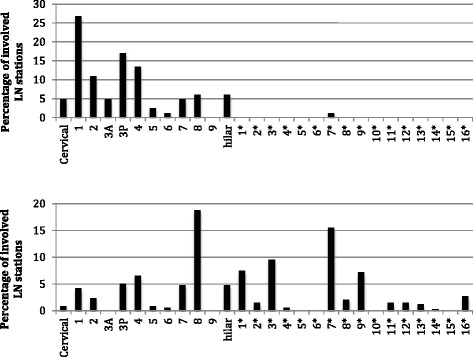
Frequency of lymph node station involvement by tumor location. Numbers with *asterisks* represent abdominal nodal stations. **a** Upper thoracic tumor (*n* = 31). **b** Lower thoracic tumor (*n* = 173)

FDG-avid LNM were identified in 204 patients (43%), with 95% having LNM in 3 or fewer stations (range 1–12, Table 2). LNM distribution and longitudinal distances from tumor are presented in Tables 3, 4, 5 and Fig. 1 below.

**Table 2:** Number of involved lymph node stations per patient

*n*	Frequency	%
0	270	57
1	113	24
2	42	9
3	25	5
4	8	2
5	6	1
6	2	0
7	1	0
8	3	1
9	2	0
10	1	0
12	1	0

**Table 3:** Longitudinal distance from primary tumor to paraesophageal lymph node metastases (thoracic stations 1 (supraclavicular), 2 (upper paratracheal), 3P (retrotracheal), 4 (lower paratracheal), 7 (subcarinal), and 8 (paraesophageal, below the carina)). Note that median distance is 0 for upper thoracic tumors due to most such lymph nodes being adjacent to primary tumor

Tumor location	Median (cm)	Range (cm)
Upper thoracic	0	0–5.3
Lower thoracic	1.52	0–18.5

**Table 4:** Percentage of all involved thoracic lymph node stations in upper vs. lower esophageal cancers. (i.e., cervical nodes represented 5% of the total number of FDG-avid nodal stations in upper thoracic tumors)

	Tumor location	
Thoracic nodal stations	Upper thoracic	Lower thoracic/GE junction
Cervical	5	1
1 (Supraclavicular)	27	4
2 (Upper paratracheal)	11	2
3A (Prevascular)	5	0
3P (Retrotracheal)	17	5
4 (Lower paratracheal)	13	7
5 (Aortopulmonary window)	2	1
6 (Para-aortic)	1	1
7 (Subcarinal)	5	5
8 (Paraesophageal, below carina)	6	19
9 (Pulmonary ligament)	0	0
10, 11 (hilar)	6	5

**Table 5:** Percentage of all involved abdominal lymph node stations in upper vs. lower esophageal cancers. (i.e., cervical nodes represented 5% of the total number of FDG-avid nodal stations in upper thoracic tumors)

	Tumor location	
Abdominal nodal stations	Upper thoracic	Lower thoracic
1 (Right paracardial)	0	8
2 (Left paracardial)	0	2
3 (Lesser curvature)	0	10
4 (Greater curvature)	0	1
5 (Suprapyloric)	0	0
6 (Infrapyloric)	0	0
7 (Left gastric)	1	16
8 (Common hepatic)	0	2
9 (Celiac)	0	7
10 (Splenic hilum)	0	0
11 (Splenic)	0	2
12 (Hepatoduodenal)	0	2
13 (Posterior pancreatic head)	0	1
14 (Superior mesenteric)	0	0
15 (Middle colic)	0	0
16 (Para-aortic)	0	3

### Upper thoracic tumors

UT tumors almost exclusively involved thoracic LNs, with only 1 out of the 82 LNMs seen in an abdominal station (1%). There were also a small number of patients (5% of UT and 1% of LT) with cervical LNM (i.e. above the supraclavicular region), that were nevertheless considered regional disease by the treating physicians and underwent definitive radiotherapy. The most commonly involved lymph node stations were supraclavicular (level 1, 27% of all involved LN stations in UT tumors), retrotracheal (level 3P, 17% of involved stations), lower paratracheal (level 4, 13%) and upper paratracheal (level 2, 11%). Supraclavicular (level 1) and paratracheal LNs (levels 2, 3P and 4) together accounted for 68% of all involved nodal regions in UT tumors. The vast majority of paraesophageal LNMs (89%) were at the same axial level as the primary tumor (i.e. visible on the same axial CT slice as the primary), and none were >5.3 cm away.

### Lower thoracic tumors

LT tumors showed a much wider distribution of LNM across thoracic and abdominal LNs. The most commonly involved lymph node stations were paraesophageal below the carina (level 8, 19% of involved stations) and those in the gastrohepatic space, including right paracardial (level 1), lesser curvature (level 3), and left gastric nodes (level 7), together comprising 34% of involved nodes in LT tumors. The remainder of involved nodes were distributed among all but 7 of the 28 thoracic and abdominal node stations surveyed. Longitudinal distances from LT tumors to paraesophageal LNMs were greater than for UT, with 58% of paraesophageal LNMs non-adjacent to the primary tumor. Unlike upper thoracic tumors, non-adjacent paraesophageal LNMs were likely to be far from the primary tumor, with 24% being over 8 cm away (Tables 3, 4, 5 and Fig. 1).

## Discussion

Guidelines for elective nodal irradiation (ENI) in esophageal cancer were originally developed in an era of two-dimensional treatment planning, and are therefore still based on simple geometric expansions of the primary tumor volume rather than cross-sectional nodal anatomy. Moreover, these geometric expansions are relatively large, given the propensity of esophageal cancer to have occult involvement of regional nodes or to recur in those areas after local therapy.

With the routine use of CT and PET-CT imaging and the availability of conformal RT techniques such as IMRT, radiation oncologists now have the ability to delineate and selectively deliver RT to CTVs based on cross-sectional nodal anatomy. However, data is needed to guide CTV delineation with reference to cross-sectional imaging in order to maximize the probability that elective nodal irradiation will be effective in reducing locoregional recurrence. An esophageal contouring atlas, based on expert consensus, has recently been published and provides guidelines for ENI based upon the data available, while acknowledging the need for additional data and the limitations inherent in trying to generalize the available data to CTV delineation [14]. An additional CTV atlas, specific to squamous cell carcinoma of the thoracic esophagus, has also recently been published [15].

Generally, current clinical practice is to expand the CTV approximately 3–4 cm longitudinally and 1–2 cm radially from the GTV as noted above. The practice-defining CROSS trial, for example, specified a 4 cm longitudinal PTV expansion from tumor, which corresponds to a 3 cm CTV expansion assuming a typical 1 cm PTV expansion from CTV [16]. In addition, many practitioners further extend the CTV inferiorly to cover the celiac axis nodes for LT tumors, and to the supraclavicular basins for UT tumors, further expanding the size of the irradiated field relative to the tumor. In the case of a distal or GE junction cancer, it is not uncommon for the CTV to be 10–20 times as large as the GTV, after standard expansions have been applied. Greater CTV volumes will inevitably lead to greater volumes of irradiated normal tissues, including the heart, lungs, stomach, bowel, and esophagus itself, which in turn leads to a greater probability for acute and late toxicity. Therefore, if low-risk nodal regions can be safely excluded from the CTV, the therapeutic ratio for RT should increase.

A number of studies report on the pattern of LNM in esophageal cancer, based on surgical pathologic data. Two studies from China represent the most significant efforts to map LNM, based on surgical pathologic data from patients with thoracic esophageal SCC [6, 17]. However, those results are less relevant to current practice in the West, where adenocarcinomas arising from the distal esophagus or GE junction comprise the majority of EC. A small number of studies have reported pathologic patterns of lymph node involvement after surgical resection of GE junction adenocarcinoma [18–20]. These studies are generally concurrent with our findings that for distal esophageal and GE junction cancers, the gastrohepatic space and paraesophageal nodes are most commonly involved, and that for UT tumors, metastasis rates to lower mediastinal and abdominal nodes are low.

Our study expands the knowledge base for patterns of nodal involvement in a number of ways, however: it is based on a significantly larger pool of patients than either the Meier or Dresner studies (and hence is the largest published dataset of nodal involvement for esophageal adenocarcinoma, to our knowledge). We also suggest that PET-CT-based data can be more readily extrapolated to radiation oncology planning, which is a CT-based endeavor. For example, determining the radiographic distance between GTV and paraesophageal nodes cannot be accurately measured from pathologic data due to artifacts induced by histopathologic processing and the inability to precisely preserve and record precise anatomic relationships that existed prior to surgery.

The results of this study provide information on the potential distribution of LNM in esophageal cancer by describing the patterns of FDG-avid LNM at the time of presentation. Though the purpose of ENI is to reduce nodal relapse by targeting potential occult disease, it is highly likely that the pattern of occult disease conforms to the patterns of initial FDG-avid nodal disease described in this study. To a large extent, our results confirm the prevailing guidelines for elective nodal coverage. With upper esophageal cancers, for example, we found a high rate of supraclavicular nodal involvement. This supports the routine inclusion of the supraclavicular nodes in the CTV for tumors arising above the level of the carina, despite other studies suggesting no clear role for elective nodal radiation of any kind in squamous esophageal cancer. Uncertainty persists regarding the need for inclusion of the anterior mediastinal nodes (levels 3A, 5, and 6) in the CTV, however. We identified some patients with FDG-avid anterior mediastinal nodes, but collectively they represented less than 10% of upper esophageal cases.

Our data is unique in allowing us to analyze patterns of LNM across multiple histologies and subsites of esophageal cancer, in contrast to the cited studies that were limited to squamous cell cancers or GE junction cancers exclusively. We found that the distribution of LNM was much more circumscribed for upper esophageal cancers, compared to distal or GE junction cancers. Since it was rare for upper esophageal cancers to metastasize to distant para-esophageal nodes, this study suggests that there is no need for large CTV expansions inferiorly below the level of the tumor.

The majority of our study population comprised lower esophageal and GE junction adenocarcinomas, and further insights can be gleaned from our analysis of this population. Generally, these patients demonstrated a greater distribution of LNM among both thoracic and abdominal stations. Since most distal tumors are adenocarcinomas, this is also consistent with the notion that adenocarcinomas have a more widespread distribution of potential recurrence compared to squamous cell carcinomas. Our data confirms a significant rate of paraesophageal LNM, which supports the existing guidelines for radial CTV expansions from tumor and esophagus. We also found clear patterns in abdominal LNM, which is informative, as traditional guidelines for esophageal cancer CTV typically do not define the extent of recommended abdominal LN coverage in terms of defined nodal stations. In particular, it was very common for LNM to arise in the gastrohepatic space (which correspond to the contiguous stations 1, 3 and 7 in the Japanese Research Society for the Study of Gastric Cancer classification). Therefore, we would strongly recommend the routine inclusion of these nodal regions in the CTV for distal esophageal cancers.

On the other hand, we did not identify a high rate of involvement of the splenic hilar or greater curvature nodes, which have sometimes been suggested as candidates for inclusion in the CTV for GE junction tumors [19]. Since inclusion of these nodes significantly increases the CTV size, and particularly the dose to radiosensitive structures such as the stomach and kidney, our data provides some reassurance that omission of those nodal sites should not lead to an excessive rate of disease recurrence in those sites. It should be noted, however, that tumors with significant cardia involvement would have been classified as primary gastric cancers and therefore excluded from this study.

Another area of controversy is the necessity for celiac nodal coverage, particularly since this is the most caudal region considered in esophageal cancer RT and therefore results in significant enlargement of the CTV. A report from the MD Anderson Cancer Center indicated that the rate of celiac nodal recurrence, though not especially common, appeared high enough to warrant elective coverage [21]. Though we found that the celiac nodes were not among the most commonly involved nodes in distal esophageal tumors, we still observed a high enough rate that we likewise suggest it be strongly considered for routine inclusion in the CTV.

Finally, we note that the relationship of paraesophageal LNM to the primary tumor was not predictable, and that such LNM could arise far from the primary tumor. The implications of this observation are not straightforward. Very large longitudinal CTV expansions could be used to cover occult paraesophageal nodal involvement. However, the potential toxicity of radiating such a substantial length of the thorax and esophagus is high. Therefore, even though large longitudinal CTV expansions may treat additional sites of LNM, we conjecture that the therapeutic ratio of radiotherapy might be maximized by limiting longitudinal CTV to what is required to encompass the primary tumor alone. The question of optimal longitudinal CTV expansions deserves further study.

The major limitation of this study is the ability of PET-CT to accurately identify metastatic lymph nodes. In general, because FDG-PET only identifies macroscopic lymph node disease, PET-based data will understate the actual rates of pathologic LN involvement. For this reason, the rates of nodal involvement reported here cannot be directly compared to rates derived from pathologic data. Previous meta-analyses have placed the sensitivity and specificity of detecting involved lymph nodes in esophageal cancer with ^18^FDG PET-CT between 55–62% and 76–96% respectively [22, 23]. Other studies have reported varying accuracies depending on tumor type, stage, SUV max, and the location of lymph node involvement [10, 24–26]. One reason for the relatively lower sensitivity of PET is its limited spatial resolution, which can make it difficult to distinguish paraesophageal LNM from adjacent FDG-avid primary tumor.

Though PET-CT alone may not fully reflect the true nodal stage in an individual patient, our aggregated PET-based data are still relevant towards the study goal, which is to identify the regional nodes at highest relative risk of involvement for esophageal cancer patients as a group. Though patterns of locoregional relapse (which are most likely correlated to occult microscopic disease) may not mirror the patterns of gross FDG-avid nodal involvement described here, it is likely that there is some correlation, and we present this data as a supplement to existing literature on pathologic nodal involvement and observed sites of locoregional recurrence. And the fact that PET underestimates the rate of GTV-adjacent LNM is less relevant to the question of optimal CTV delineation, since GTV-adjacent LNM will always be covered as long as there is sufficient margin on the GTV itself. With respect to identifying high-risk regional nodes that would not otherwise be covered with margin on GTV, our data should be of significant utility.

Overall, this analysis provides a basis for individualized CTV delineation based on the location of the primary tumor, and with reference to radiographic nodal stations rather than standard geometric expansions or bony anatomy. We propose that the CTV for upper thoracic tumors routinely include the bilateral supraclavicular basins and the paraesophageal nodes at the level of the FDG-avid tumor, but not significantly below. The CTV for lower thoracic and GE junction tumors should always include the lymph nodes in the gastrohepatic space, and likely also the celiac region, in addition to paraesophageal nodes at the level of FDG-avid tumor. Though distal esophageal adenocarcinomas have the potential to spread to multiple other lymph node regions throughout the abdomen and thorax, it does not appear to do so in a regular or frequent enough fashion to propose routine inclusion of other nodal regions besides the ones already mentioned.

These data and the resulting guidelines, being based on retrospective data from a single-institution series, obviously cannot be considered authoritative. However, they are derived from a significant number of patients and largely concur with what has been suggested by existing literature. Similar analyses of other patient cohorts, particularly at institutions or regions with different mixes of esophageal cancer histologies and presentations, will help to strengthen or refine these conclusions.

## Conclusions

A more data-driven and individualized approach to CTV delineation could improve the therapeutic ratio of RT in esophageal cancer. These results can guide CTV delineation by indicating the potential distribution of nodal involvement in esophageal cancer. Our data identified clear patterns in abdominal LNM, with frequent involvement of gastrohepatic and celiac, but not splenic hilar or greater curvature nodes. Additionally, our study confirms a significant rate of LNM directly adjacent to paraesophageal tumors, especially those above the carina. However, the distance between paraesophageal tumors and LNM was not predictable, suggesting that large longitudinal CTV expansions to cover occult LNM may not always be warranted.

## Additional file

Additional file 1: Deidentified patient data. (XLSX 145 kb)

## Abbreviations

CTV: Clinical target volume
EC: Esophageal cancer
ENI: Elective nodal radiation
FDG: Fludeoxyglucose (^18^F)
GE: Gastroesophageal
GTV: Gross tumor volume
IMRT: Intensity-modulated radiation therapy
LNM: Lymph node metastasis
LT: Lower thoracic
PET: Positron emission tomography
PET-CT: Positron emission tomography-computed tomography
RT: Radiation treatment
SCC: Squamous cell carcinoma
UT: Upper thoracic
